# Study on the Impact of Pea Protein on the Printability and Storage Quality of the 3D Printing Pork Jerky

**DOI:** 10.3390/foods14213701

**Published:** 2025-10-29

**Authors:** Ligen Wu, Anna Wang, Qihan Cui

**Affiliations:** 1 College of Food Science and Engineering, Henan University of Technology, Zhengzhou 450002, China; ligen2016@126.com (L.W.); wds7002@163.com (Q.C.); 2 National Engineering Research Center of Wheat and Corn Further Processing, Henan University of Technology, Zhengzhou 450002, China

**Keywords:** 3D printing, pork products, storage quality, Maillard reaction products, HS-SPME-GC-MS (headspace solid-phase microextraction gas chromatography-mass spectrometry)

## Abstract

Food 3D printing technology for meat products has garnered significant attention. However, natural meat often requires the modification of its relevant properties to meet the processing demands of 3D printing. This study utilized minced pork as the primary raw material and employed pea protein to enhance both the 3D printability and storage quality characteristics of the pork paste. The results indicated that the optimal printing performance was achieved with a pea protein addition level of 20% (pork-to-pea protein mass ratios of 5:1). Specifically, pea protein significantly improved the material properties critical for 3D printing, enhancing the gel strength rising from 93.31 to 241.52 g and stability of the printing material. The 3D-printed pork products were stored at 25, 35, and 45 °C for 44 days, with increasing storage temperature, the moisture content, water activity, pH, and freshness of the 3D-printed meat products significantly decreased (*p* < 0.05), after 30 days of storage at 45 °C, the acid value reached 4.13 mg/g. During storage, a total of 233 volatile flavor compounds were identified, which comprised 17 esters, 26 alcohols, 58 terpenes, 69 alkanes, 20 aldehydes, 29 heterocyclic compounds, and 14 other compounds (including acids, ketones, and ethers), among 19 key flavor compounds, nonanal, phenethylaldehyde, D-limonene, zingiberene, and α-curcumene contributed significantly to the flavor profile of the pork jerky. Elevated storage temperatures and time leading to a notable deterioration in the storage quality of the 3D-printed pork products. The shelf life of 3D-printed pork products, when stored without preservatives, is limited to 44 days at a recommended maximum temperature of 35 °C.

## 1. Introduction

Recent advances in food 3D printing have demonstrated substantial capabilities in personalized nutrition and intricate culinary design [1,2,3]. While applications utilizing animal-derived materials such as pork, chicken, shrimp, and beef remain prevalent [4,5,6], emerging research highlights the technology’s potential to redefine custom food fabrication [7]. However, research on 3D printing with pork is relatively scarce, Guan et al. (2025) [8] successfully engineered structured streaky pork by employing a novel strategy of 3D co-printing and co-differentiation of porcine muscle stem cells and porcine adipose-derived mesenchymal stem cells. Xu et al. (2023) [9] evaluated the effect of xanthan gum (XG) on the rheological properties and 3D printability of pork pastes. Food 3D printing is an additive manufacturing process whereby edible materials are deposited layer-by-layer onto a substrate following digitally designed paths, ultimately solidifying into three-dimensional structures [10]. This technology imposes specific rheological requirements on printing materials to ensure structural integrity and self-supporting capacity during deposition [11]. Unmodified natural food ingredients often lack these functional attributes, thus requiring tailored modifications to achieve printability. Previous studies have employed hydrocolloids, carbohydrates, lipids, and protein-based additives to modulate the rheological properties of meat paste, thereby enhancing the printability of native meat materials for 3D printing applications [12,13,14,15]. Pea protein, characterized by its hypoallergenic properties and well-balanced amino acid profile with high lysine content [16], exhibits exceptional functional attributes including water-/oil-binding capacity, emulsification, and gelation. These properties enable its application as a functional adjuvant in food 3D printing. Empirical studies by Feng et al. (2018) [17] and Álvarez-Castillo et al. (2021) [18] demonstrate its efficacy in enhancing print fidelity in potato starch-based matrices and chicken paste systems, respectively.

Current investigations into 3D printed food are largely driven by efforts to optimize the fabrication technology and to enhance the understanding of the behavior and composition of food inks [19], there is a notable absence of large-scale trials in the literature that examine changes in the quality attributes of 3D-printed foods during storage [19]. The integration of 3D printing technology into meat product fabrication presents unique challenges for maintaining sensory quality during storage, particularly concerning flavor stability. Storage leads to a decline in desirable alcohols and an increase in aldehydes and acids, which can negatively impact the overall flavor acceptance and introduce increased sourness. Flavor represents a critical determinant of meat product quality, originating primarily from protein degradation, lipid hydrolysis/oxidation, and Maillard reactions, yielding volatile compounds including ketones, aldehydes, hydrocarbons, alcohols, esters, acids, and furans [20]. These molecules constitute the principal sources of lipid-derived aroma compounds. Monitoring the compositional and quantitative dynamics of volatile flavor compounds during storage provides a robust analytical framework for evaluating flavor evolution in 3D-printed meat products [21].

This study addresses these critical knowledge gaps by examining the influence of pea protein supplementation on the printability and the physicochemical quality, without the microbiological or sensorial quality during storage of 3D-printed pork jerky, thereby providing a hypothesis for explaining the role of pea protein.

## 2. Materials and Methods

### 2.1. Material and Reagents

Deboned pork hindquarter cuts was procured from a local supermarket in the Henan Province, Zhengzhou, China.

Pea protein (food grade, soy protein isolate, protein content of over 90% on a dry basis, with a moisture content of less than 7%) was supplied by Henan Wanbang Biological Technology Co., Ltd. (Shangqiu, China). The following reagents were obtained from commercial suppliers: potassium bromide (spectrophotometric grade) and fluorescein isothiocyanate (spectrophotometric grade) from Tianjin Komio Chemical Reagent Co., Ltd. (Tianjin, China); thymolphthalein indicator and sodium thiosulfate (analytical grade) from Shanghai Macklin Biochemical Technology Co., Ltd. (Shanghai, China); potassium iodide (analytical grade) from Tianjin Jinbei Fine Chemical Co., Ltd. (Tianjin, China); 2-thiobarbituric acid (analytical grade), trichloroacetic acid (analytical grade), and magnesium oxide (analytical grade) from Macklin Biochemical Technology Co., Ltd. (Shanghai, China); petroleum ether (analytical grade) from Tianjin Jindong Tianzheng Fine Chemical Reagent Factory (Tianjin, China).

### 2.2. 3D Printing Process

Deboned pork hindquarter cuts were manually trimmed to remove skin and connective tissues. Lean and adipose tissues were combined at a ratio of 8.5:1.5 (*w*/*w*) and comminuted using a mechanical grinder to achieve a homogeneous paste consistency.

For pork-pea protein printing formulations, five mass ratios of pork to pea protein were evaluated: 2:1, 3:1, 4:1, 5:1, and 6:1. All formulations were supplemented with the following additives relative to total mass: 20% sucrose, 6% fish sauce, 1% glycerol, 0.2% black/white pepper blend, 0.2% ginger powder, 0.15% compound moisture retention agent, 0.1% sodium D-isoascorbate, 2% NaCl, and 35% deionized water. The mixtures were homogenized using a high-shear mixer at 4 °C for 15 min and subsequently conditioned at 4 °C for 10 h prior to printing.

### 2.3. 3D Model Design and Printing Parameters

A rectangular prism (50 × 40 × 3 mm) was designed using computer-aided design (CAD) software to simulate the dimensional characteristics of traditional pork jerky. The model was sliced using Simplify3D software (v.4.1.2) with the following printing parameters: nozzle temperature 25 °C, nozzle diameter 1.55 mm, 100% infill density, and printing speed 30 mm/s, using the FOOD BOT Food 3D Printer (Hangzhou Shiyin Technology Co., Ltd., Hangzhou, China).

### 2.4. Thermal Processing of 3D-Printed Pork Products

The 3D-printed meat specimens were subjected to convective drying in a hot-air oven at 55 °C for 5 h. Subsequently, the dehydrated matrices were cooled to ambient temperature (25 ± 2 °C) and baked in a preheated conventional oven at 220 °C for 3 min. The thermally treated products were immediately flattened under uniform pressure and allowed to equilibrate to room temperature prior to vacuum packaging in bags (Composite Aluminum Foil Bag (PET Matte 15 μm/PA 15 μm/AL 7 μm/PA 15 μm/PE 70 μm)) [22].

### 2.5. Storage Conditions for 3D-Printed Pork Products

Vacuum-packaged samples were subjected to accelerated storage testing in precision incubators maintained at 25 °C, 35 °C, and 45 °C. Sampling was performed at designated time points (0, 3, 6, 9, 16, 23, 30, 37, and 44 days) for subsequent analytical evaluations throughout the 44-day storage period.

### 2.6. Determination of Moisture, pH, Total Volatile Basic Nitrogen, Acid Value, and Peroxide Value

Moisture content was determined according to GB/T 5009.3-2016 (Direct drying method for determination of moisture in foods) [23]. Water activity was measured following GB/T 5009.238-2016 (Determination of water activity in foods—Method II) [24]. The pH values were measured in accordance with GB/T 5009.237-2016 (Determination of pH in foods) [25]. Total volatile basic nitrogen (TVB-N) was determined using GB/T 5009.228-2016 (Determination of volatile basic nitrogen in foods—Automatic Kjeldahl nitrogen analyzer method) [26]. Acid value was analyzed according to GB/T 5009.229-2016 (Determination of acid value in foods—Method I) [27]. Peroxide value was determined following GB/T 5009.227-2023 (Determination of peroxide value in foods—Indicator titration method) [28].

### 2.7. Determination of TBARS and Maillard Reaction Products

The Thiobarbituric Acid Reactive Substances (TBARS) assay was performed according to the method described by Qiu et al. (2023) [29] with modifications. Briefly, 2 g of sample was homogenized with 20 mL of trichloroacetic acid (TCA) aqueous solution using a vortex mixer for 1 min. The mixture was allowed to stand at room temperature for 30 min, followed by centrifugation at 4000 r/min for 10 min. The supernatant was filtered through qualitative filter paper. Subsequently, 5 mL of the filtrate was mixed with 5 mL of TBA solution (0.02 mol/L) by vortexing for 30 s. The reaction mixture was heated in a water bath at 90 °C for 30 min and immediately cooled in an ice-water bath to room temperature. The absorbance of the resulting solution was measured at 532 nm using a UV7600 double beam UV-visible spectrophotometer (Shanghai Lengguang Technology Co., Ltd., Shanghai, China). A blank solution containing 5 mL TCA solution mixed with TBA solution was used for zero adjustment. A standard curve was established using 1,1,3,3-tetraethoxypropane as the standard, yielding the regression equation y = 1.1922x + 0.0101 (R^2^ = 0.9998).

Maillard reaction products (MRPs) were quantified based on the protocol established by Zhang et al. (2024) [30] and Han et al. (2020) [31] with modifications. Briefly, 3 g of pulverized 3D-printed pork sample was homogenized with 30 mL of trichloroacetic acid (TCA, 20% *w*/*v*) using vigorous vortexing for 2 min. The mixture was allowed to stand for 10 min at room temperature, followed by centrifugation at 8000 r/min for 10 min. The supernatant was filtered through qualitative filter paper and subsequently analyzed using a UV7600 double beam UV-visible spectrophotometer (Shanghai Lengguang Technology Co., Ltd.) to measure absorbance at 294 nm (intermediate MRPs) and 420 nm (brown pigments).

### 2.8. Rheological Characterization

Rheological properties were determined according to the method described by Pan et al. (2021) [32] with modifications. Measurements were conducted using the HAAKE MARS60 rheometer from Thermo Fisher Scientific Inc. (Waltham, MA, USA) with a P35 rotor system in a 1 mm plate gap. The strain amplitude was set at 0.5% to ensure measurements remained within the linear viscoelastic region. All tests were performed at 25 °C with dynamic frequency sweeps conducted over an angular frequency range of 0.1–20 Hz.

### 2.9. Low-Field Nuclear Magnetic Resonance (LF-NMR) Analysis

Samples of 3D-printed pork products were wrapped in plastic film and positioned within the instrument chamber for analysis using the IIVTMR 20-010 low-field NMR analyzer from Suzhou Niumag analytical instrument corporation (Suzhou, China). The following parameters were employed for water distribution assessment: sampling frequency of 200 kHz, wait time of 2000 ms, echo time of 0.1 ms, 15,000 echoes, and 16 scan accumulations.

### 2.10. Gel Strength Determination

Gel strength was evaluated on the TA-XT Plus texture analyzer from the stable micro systems (SMS) Ltd., UK (London, UK) using the following parameters: Return to start mode with a P0.5 probe, pre-test speed of 2 mm/s, test speed of 1 mm/s, post-test speed of 2 mm/s, trigger force of 5 g, and compression distance of 10 mm.

### 2.11. Confocal Laser Scanning Microscopy (CLSM)

Samples of 3D-printed pork product (1 g) were stained with 200 μL fluorescein isothiocyanate (FITC) solution and thoroughly homogenized. The mixture underwent light-protected incubation at 25 °C for 30 min. Subsequently, an aliquot of the stained sample was mounted under a coverslip for microscopic observation. CLSM imaging was performed on the FV3000 laser scanning confocal microscope (Olympus Corporation, Tokyo, Japan) using an excitation wavelength of 494 nm for FITC, with emission detection in the 500–540 nm range.

### 2.12. Headspace Solid-Phase Microextraction Gas Chromatography-Mass Spectrometry (HS-SPME-GC-MS)

Volatile compounds were analyzed with the trace 1300-ISQ 7000 gas chromatograph-mass spectrometer (Thermo Fisher Scientific Inc., USA) following the method of Han et al. (2020) [31] with modifications. Briefly, 2 g of pulverized 3D-printed pork sample (passed through a 40-mesh sieve) was placed in a 100 mL conical flask, hermetically sealed, and equilibrated at 60 °C for 20 min prior to SPME extraction for 40 min.

GC Conditions: Separation was performed on an HP-5MS capillary column (30 m × 0.25 mm, 0.25 μm) with helium (purity > 99.999%) as carrier gas at 1.0 mL/min. The injector temperature was maintained at 230 °C with a 5 min thermal desorption time. Manual splitless injection was employed.

Temperature Program: Initial oven temperature 35 °C (hold 5 min), ramped to 155 °C at 3 °C/min, then increased to 230 °C at 15 °C/min (hold 5 min). Total run time: 55 min.

MS Conditions: Electron impact (EI) ionization at 70 eV; ion source temperature 230 °C; mass scan range *m*/*z* 35–550. Relative compound abundances were quantified using peak area normalization.

Compound identification was based on three criteria: Comparison with the NIST mass spectral library, with a match factor threshold set at 800; Calculation of retention indices using a homologous series of C7-C30 n-alkanes and comparison with literature values; Confirmation of key flavor compounds such as hexanal and nonanal by comparing their retention times with those of authentic standards. Semi-quantification was performed using the internal standard method with 2-octanol as the internal standard, and the relative contents of the volatile compounds were calculated accordingly.

### 2.13. Analysis of Key Volatile Flavor Compounds

Key volatile flavor compounds were identified using the relative odor activity value (ROAV) method. Compounds with ROAV ≥ 1 were considered to contribute significantly to the overall flavor profile of 3D-printed pork products and were defined as key flavor compounds. Those with 0.1 ≤ ROAV < 1 were recognized as having a modifying role in the flavor characteristics. The ROAV was calculated as follows:

ROAV = (C_i_/C_stan_)/(T_i_/T_stan_) × 100 (1)

where C_i_ and C_stan_ represent the relative concentrations (%) of a given volatile compound and the most impactful flavor compound, respectively; Tᵢ and T_stan_ denote the odor thresholds (mg/kg) of the corresponding compounds. The odor-threshold sources cited from Van Gemert (2015) [33].

### 2.14. Statistics

Statistical analyses were performed using SPSS 20.0 (IBM Corp., Armonk, NY, USA). Significant differences among treatments were determined by one-way analysis of variance (ANOVA) followed by post-hoc tests (Tukey), Two-way ANOVA was employed to examine the significance of the effects of intergroup factors and storage time (temporal factor) on various indicators. Tukey’s HSD (or Duncan’s) multiple comparison test was applied to analyze significant differences between groups with a significance level defined at *p* < 0.05. Data visualization was conducted using Origin 2021 (OriginLab Corp., Northampton, MA, USA). All experiments were performed in triplicate, and results are expressed as mean ± standard deviation.

Pearson correlation analysis was conducted to assess the linear relationship between the measured indicators included storage temperature, storage time, moisture content, water activity, pH, color parameters (L, a, b*), TVB-N, fat content, acid value, TBARS, peroxide value (PV), and absorbance at 294 nm (A294) and 420 nm (A420). The strength and direction of the relationship were quantified using the Pearson correlation coefficient (r), which ranges from −1 (perfect negative correlation) to +1 (perfect positive correlation).

## 3. Results and Discussion

### 3.1. Effect of Pea Protein on the Storage Modulus (G′) and Loss Modulus (G″) and Gel Strength of 3D-Printed Pork Paste

**Figure 1:**
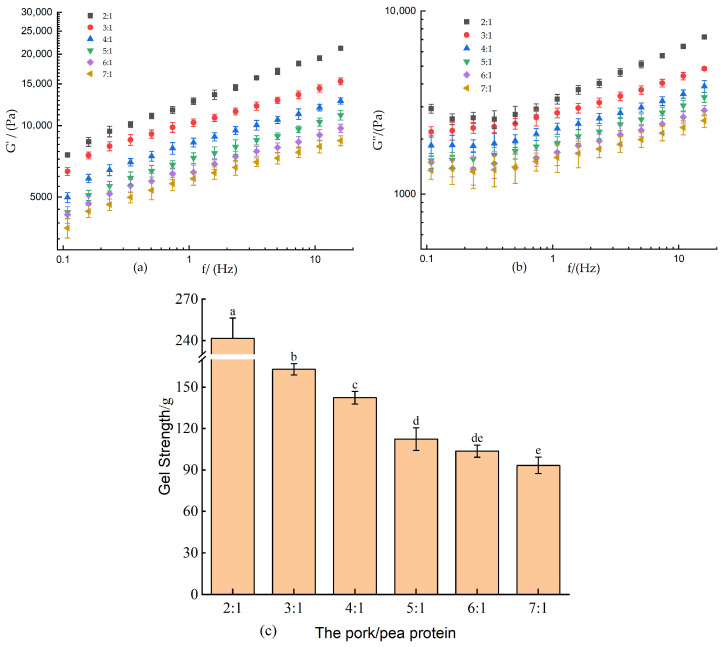
Effect of pea protein incorporation on the dynamic rheological properties and gel strength of 3D-printed pork paste. (**a**) storage modulus (G′), (**b**) loss modulus (G′′), and (**c**) gel strength. Different letters above bars indicate significant differences (*p* < 0.05) among means for the same parameter.

The storage modulus (G′) and loss modulus (G″) serve as critical indicators for quantitatively assessing the elastic and viscous characteristics of meat-derived gels [33]. During the 3D printing process, the material is subjected to both compressive and shear stresses, making the evolution of G′ and G″ essential for evaluating the structural support capacity and extrusion behavior of the ink [34]. As illustrated in Figure 1a,b, both G′ and G″ exhibited a concentration-dependent increase with higher levels of pea protein incorporation, indicating enhanced viscoelasticity of the composite pork paste. This improvement suggests that the modified material possesses a greater resistance to deformation under stress, thereby promoting printing stability.

Notably, G′ consistently exceeded G″ across all formulations, reflecting a dominant elastic response and solid-like behavior, which is indicative of a robust gel network. Such mechanical integrity is crucial for ensuring consistent layer stacking and shape fidelity during extrusion-based 3D printing. The enhanced structural stability not only improves print accuracy and final product uniformity but also mitigates the risk of structural collapse in the printed pork-based constructs.

Gel strength serves as a reliable indicator of the extrusion behavior and the ability of a printed object to retain its structural integrity after 3D printing [35]. As demonstrated in Figure 1c, the gel strength increased significantly (*p* < 0.05) with higher pea protein incorporation, rising from 93.31 to 241.52 g. This suggests that greater proportions of pea protein promote the formation of a more compact and cohesive gel network. The enhancement in gel strength may be attributed to improved hydration through interactions between water molecules and amino acid side chains of the protein, resulting in strengthened hydrogen bonding and a more rigid matrix [27]. The most pronounced improvement in gel strength was observed when the ratio shifted from 3:1 to 2:1, a trend consistent with the rheological properties. This correlation suggests a positive relationship between gel strength and the viscoelastic parameters, further supporting the role of pea protein in reinforcing the functional performance of the meat-based material for 3D printing.

### 3.2. Effect of Pea Protein on 3D Printing Performance

**Figure 2:**
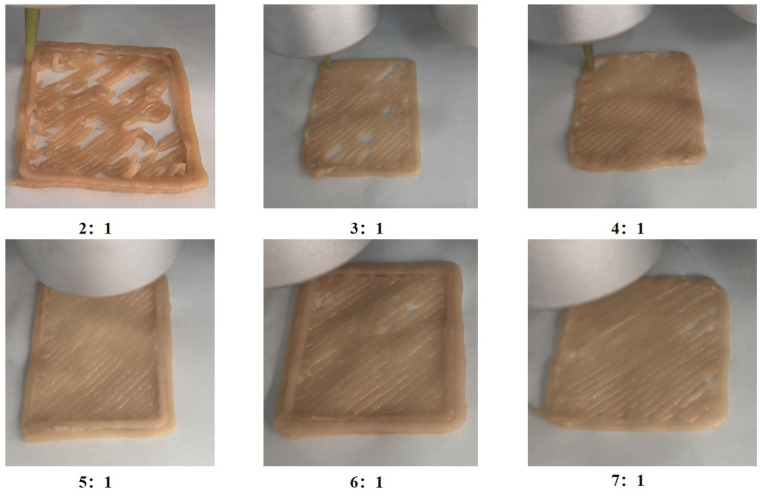
Effect of pea protein addition level on the 3D printing performance of pork paste. Note: The images correspond to mass ratios of pork to pea protein at 2:1, 3:1, 4:1, 5:1, 6:1, and 7:1, respectively.

Printing fidelity is a critical indicator for evaluating the effectiveness of 3D printing processes. As shown in Figure 2, when the mass ratio of pork to pea protein was 2:1, frequent nozzle clogging, filament breakage, and structural collapse were observed, resulting in severely impaired printability and poor morphological integrity. This can be primarily attributed to the strong hydrophilicity of pea protein, which leads to excessive water absorption and markedly elevated viscoelasticity of the printing material. At high protein concentrations, the material exhibits elevated resistance to shear, hindering smooth extrusion and leading to fractures during or after deposition, thereby compromising printability [36]. As the proportion of pea protein decreased, print quality initially improved and then gradually deteriorated. The optimal printing outcome was achieved at a ratio of 5:1, characterized by continuous extrusion, absence of clogging or breakage, and a smooth surface finish. However, at lower pea protein concentrations (6:1 and 7:1), occasional filament discontinuities occurred. This phenomenon is likely due to insufficient protein content and excessive water, which reduce viscosity and increase fluidity, thereby weakening cohesion and leading to intermittent material flow during printing [36].

### 3.3. Effect of Pea Protein on the Water Distribution of 3D-Printed Pork Products

**Figure 3:**
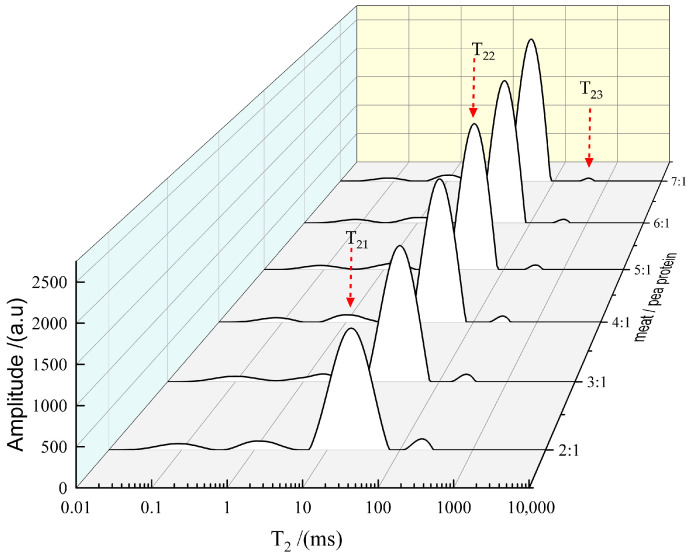
LF-NMR signal (T_2_) of mold-shaped (MS) 3D-printed pork products.

In a 3D-printed gel system, water exists in three distinct states: bound water, immobilized water, and free water (Figure 3) [37]. Therefore, the state of water distribution plays a critical role in both the printing process and the stability of 3D-printed pork products. The relaxation time T_2_ provides critical insight into the state and mobility of water in the sample. A shorter T_2_ value indicates higher stability and restricted water mobility, whereas a longer T_2_ suggests greater fluidity [38]. Specifically, the relaxation time of immobilized water (T_22_) reflects the compactness and stability of the protein network. A decrease in T_22_ is indicative of a more dense three-dimensional microstructure with enhanced water-binding capacity, which contributes to the structural integrity of the final product [39].

The incorporation of varying levels of pea protein significantly influenced both the relaxation times and relative peak areas (*p* < 0.05), as summarized in Table 1. Both T_22_ and T_23_ exhibited a prolongation as the proportion of pea protein decreased, suggesting that higher pea protein content effectively restricts water mobility. The shortest T_2_ was observed at the pork/pea protein mass ratio of 2:1, implying markedly reduced water mobility and an excessively rigid gel matrix. Although the relative peak area S_23_ was high at this ratio, the printing performance was compromised, which may be attributed to the high S_21_ and minimal T_2_, collectively pointing to severely limited water mobility that is unfavorable for 3D printing. These findings align with previous reports by Dong et al. (2020) [40] and Liu et al. (2018) [41].

### 3.4. Effect of Pea Protein on the Internal Structure of 3D-Printed Pork Products

**Figure 4:**
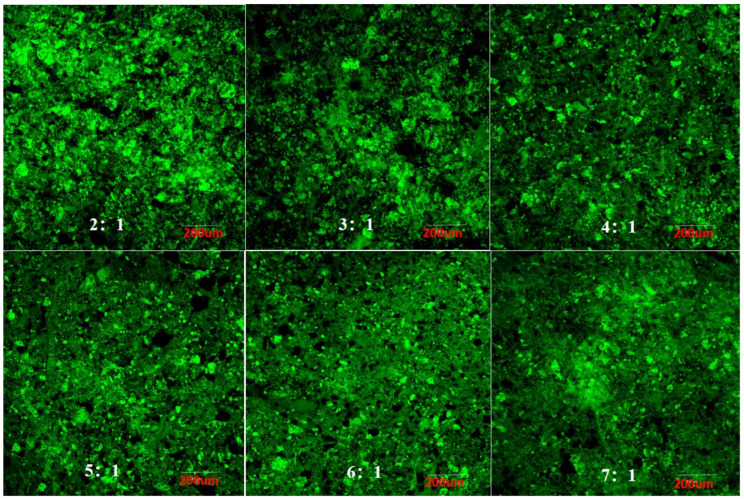
Effect of pea protein addition level on the microstructure of 3D-printed pork products. Note: The images correspond to pork-to-pea protein mass ratios of 2:1, 3:1, 4:1, 5:1, 6:1, and 7:1, respectively.

Confocal laser scanning microscopy (CLSM) enables the excitation and detection of fluorescent molecules within a sample, thereby providing detailed insights into the three-dimensional network structure of the system through fluorescence imaging [41]. As illustrated in Figure 4, at pork-to-pea protein mass ratios of 2:1 and 3:1, noticeable protein aggregation occurred within the matrix. This phenomenon can be attributed to the amphiphilic nature of pea protein, which contains both hydrophilic and hydrophobic regions. At elevated concentrations, intermolecular interactions promote self-aggregation of pea protein, leading to molecular stacking and dense cluster formation. Such structural heterogeneity adversely affects the extrusion behavior during 3D printing [41]. In contrast, at lower pea protein incorporation levels, the degree of aggregation diminished and porosity was reduced. A well-defined, homogeneous three-dimensional network was observed at the optimal ratio of 5:1, suggesting that moderate pea protein addition promotes a microstructure conducive to printability.

### 3.5. Color Changes in 3D-Printed Pork Products During Storage

Color serves as a primary visual attribute influencing consumer perception and purchasing behavior of meat products. It is quantitatively characterized by the parameters L* (lightness), a* (redness/greenness), and b* (yellowness/blueness). During storage, the surface color of the 3D-printed pork products gradually darkened, shifting from red to brown. Notably, samples stored at 25 °C and 35 °C exhibited slower color changes, retaining an acceptable reddish-brown hue even after 44 days of storage.

**Figure 5:**
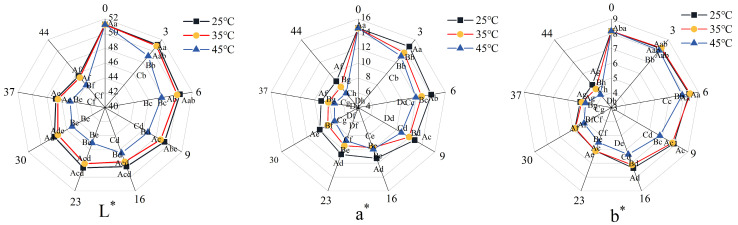
Changes in the color of 3D-printed pork products during storage. Note: L*, a*, and b* represent lightness, redness/greenness, and yellowness/blueness, respectively. Different uppercase letters indicate significant differences (*p* < 0.05) between samples stored at different temperatures at the same storage time; different lowercase letters indicate significant differences (*p* < 0.05) between samples from different storage times at the same temperature.

As shown in Figure 5, under the same storage temperature, the L*, a*, and b* values of all sample groups decreased significantly over time (*p* < 0.05). During the initial 0–6 days, the decline in L*, a*, and b* values was significantly more pronounced in samples stored at 45 °C compared to those at 25 °C and 35 °C (*p* < 0.05), while no significant differences were observed between the 25 °C and 35 °C groups (*p* > 0.05). These results indicate that storage temperatures above 35 °C exert a considerable impact on color stability, even within a short period. The color change may be attributed to the Maillard reaction and non-enzymatic browning, elevated temperatures accelerate the reactions, thereby promoting browning and leading to more rapid color deterioration in samples stored under higher temperatures compared to those under ambient conditions [42].

### 3.6. Changes in Moisture Content, Acidity, Total Volatile Basic Nitrogen, and pH of 3D-Printed Pork Products During Storage

**Figure 6:**
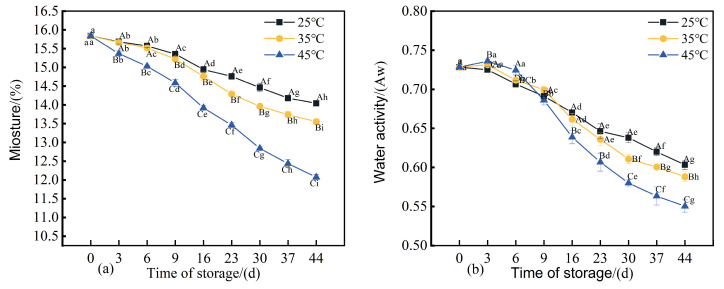

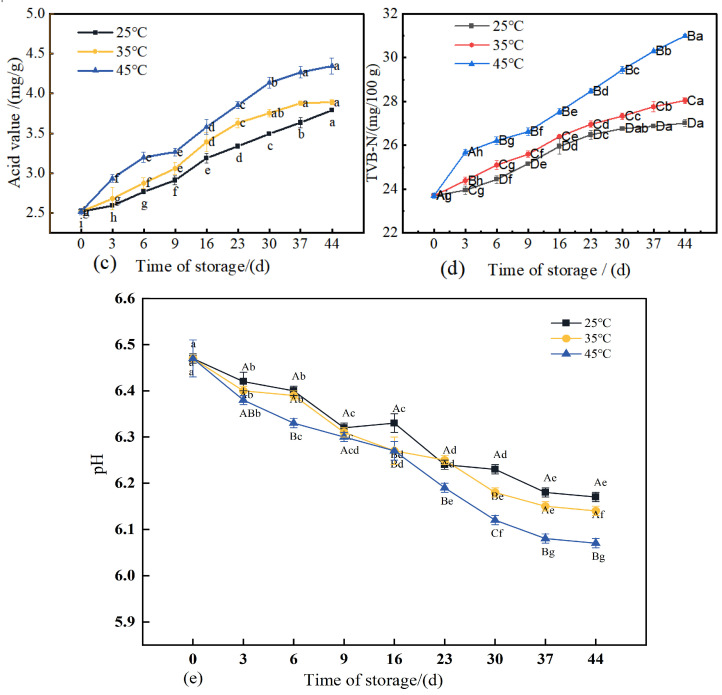
Changes in (**a**) moisture content, (**b**) water activity, (**c**) acidity, (**d**) TVB-N content, and (**e**) pH value of 3D-printed pork products during storage. Different uppercase letters indicate significant differences (*p* < 0.05) between samples stored at different temperatures at the same storage time; different lowercase letters indicate significant differences (*p* < 0.05) between samples from different storage times at the same temperature.

Water content exerts a direct influence on the texture, storage stability, and microbial growth rate of 3D-printed pork products [30]. As illustrated in Figure 6a, the moisture content of all three sample groups exhibited a declining trend over the storage period. After 44 days of storage, the moisture contents measured 14.04%, 13.55%, and 12.07%, respectively, which were significantly lower (*p* < 0.05) than the initial value of 15.8%. During the first 6 days of storage, no significant difference (*p* > 0.05) was observed between the samples stored at 25 °C and those at 35 °C, although both were significantly lower (*p* < 0.05) than the initial moisture content. This indicates that within storage temperatures ≤ 35 °C, the duration of storage exerts a greater impact on moisture loss than temperature does. Elevated storage temperatures combined with extended durations resulted in reduced water-holding capacity of the 3D-printed pork products, thereby leading to increased moisture loss. Water activity (aw) was also considerably affected by storage conditions (Figure 6b). By day 44, the water activity of all three groups had decreased significantly (*p* < 0.05) compared to the initial level, with longer storage correlating with reduced aw values.

Variations in pH exert a considerable influence on the quality of matured meat products. An appropriate pH level not only helps preserve the desired flavor profile but also inhibits microbial growth. However, an excessively low pH may lead to nutrient loss and adversely affect both sensory properties and product safety. As depicted in Figure 6e, the pH of 3D-printed meat samples stored at each of the three temperatures demonstrated a significant decreasing trend (*p* < 0.05). This decline is primarily attributed to the accumulation of acidic compounds resulting from protein hydrolysis (generating carboxyl groups), lipid oxidation, and degradation of carbohydrates into organic acids during storage, collectively contributing to a reduction in pH [43]. The acidity serves as a key indicator of the extent of lipid hydrolysis in meat products. Free fatty acids released through hydrolysis act as important precursors for flavor compounds [44]. As shown in Figure 6c, the acidity of 3D-printed pork products increased significantly (*p* < 0.05) with prolonged storage under all three temperature conditions (25 °C, 35 °C, and 45 °C). Higher storage temperatures corresponded to greater acidity, a phenomenon likely driven by enhanced activity of lipolytic enzymes at elevated temperatures, which accelerates lipid hydrolysis [45]. This suggests that increased lipid oxidation promotes the generation of free fatty acids in the 3D-printed pork. After 30 days of storage at 45 °C, the acidity reached 4.13 mg/g.

During storage, proteins in meat products undergo partial degradation, generating alkaline nitrogenous compounds such as ammonia and amines. Total Volatile Basic Nitrogen (TVB-N) serves as an indicator for assessing the freshness of meat products throughout storage [46]. As shown in Figure 6d, the TVB-N values of all three groups of 3D-printed pork products exhibited a significant increasing trend (*p* < 0.05) over time. Higher storage temperatures were associated with a more rapid accumulation of TVB-N. By the end of the 44-day storage period, the TVB-N values of the three sample groups had reached 113.9%, 118.3%, and 130.7% of their initial levels, respectively. These results demonstrate that elevated storage temperatures accelerate the production of TVB-N, leading to a more pronounced increase in its value [47].

### 3.7. Changes in Peroxide Value, TBARS, and Maillard Reaction Products in 3D-Printed Pork Products During Storage

**Figure 7:**
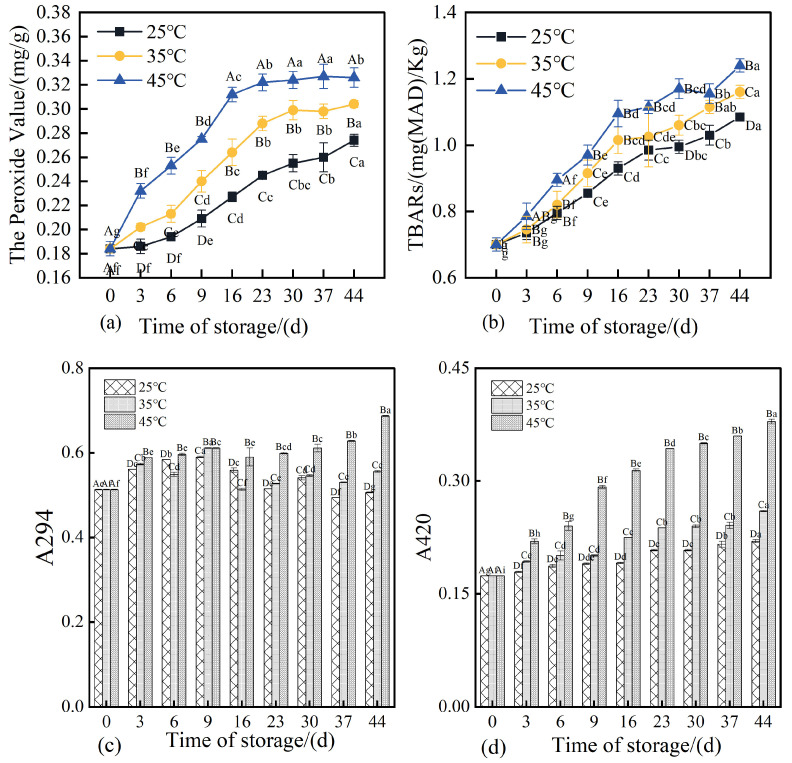
Evolution of POV, TBARS and Maillard reaction products in 3D-printed pork products during storage. Note: (**a**) Peroxide value (POV). (**b**) Thiobarbituric acid reactive substances (TBARS) value. (**c**) Absorbance at 294 nm (A_294_), indicating the concentration of intermediate Maillard reaction products. (**d**) Absorbance at 420 nm (A_420_), representing the concentration of brown polymeric melanoidins formed during the advanced stages of the Maillard reaction. Different uppercase letters indicate significant differences (*p* < 0.05) between samples stored at different temperatures at the same storage time; different lowercase letters indicate significant differences (*p* < 0.05) between samples from different storage times at the same temperature.

Peroxide value (PV) serves as a key indicator for assessing the extent of lipid oxidation, with higher PV values typically reflecting advanced oxidative degradation of lipids [48]. As illustrated in Figure 7a, the initial PV of the 3D-printed pork products was 0.184 mg/g on day 0. After 44 days of storage, the PVs of the three groups increased significantly (*p* < 0.05), reaching 0.274, 0.303, and 0.306 mg/g, respectively. This demonstrates that elevated storage temperatures accelerated the rate of lipid oxidation, with higher temperatures corresponding to more rapid increases in PV [49].

Thiobarbituric acid reactive substances (TBARS) serve as an important indicator for evaluating the extent of secondary lipid oxidation in pork jerky. Higher TBARS values reflect increased malondialdehyde (MDA) content, suggesting advanced stages of lipid oxidation [50]. As shown in Figure 7b, the TBARS values of 3D-printed pork products increased to varying degrees over storage time across all three temperature conditions, indicating an accumulation of secondary lipid oxidation products during storage. The rate of increase in TBARS was more pronounced at higher storage temperatures, with statistically significant differences observed among groups (*p* < 0.05). These findings align with those reported by Liu et al. (2024) [51], which noted an accelerated lipid oxidation rate when storage temperatures exceeded 40 °C. This phenomenon may be attributed to enhanced reactions between malondialdehyde and amino groups in proteins, as well as the degradation of MDA into smaller molecular species such as alcohols and acids due to its inherent instability [52].

The Maillard reaction in meat products is a chemical process involving carbonyl and amino compounds that undergo a series of reactions including dehydration, condensation, degradation, and polymerization, ultimately leading to the formation of compounds with distinct sensory and chemical properties [53]. The absorbance at 294 nm (A_294_) is commonly used to monitor intermediate Maillard reaction products, particularly compounds containing aromatic rings, whose presence increases UV absorption. Meanwhile, absorbance at 420 nm (A_420_) reflects the concentration of brown pigments formed in the advanced stages of the Maillard reaction, including melanoidins and other glycation products that contribute to yellow-brown coloration [30]. Under uncontrolled conditions, Maillard reaction end-products may adversely affect product quality and have been associated with potential health implications, including accelerated aging. Therefore, it is essential to investigate the progression and extent of the Maillard reaction in 3D-printed pork products during storage. As shown in Figure 7c, after 44 days of storage, the A_294_ values of samples stored at 35 °C and 45 °C increased significantly (*p* < 0.05), reaching 108% and 134% of the initial value, respectively. Higher storage temperatures resulted in greater A_294_ values, with significant differences observed among groups (*p* < 0.05). The A_294_ value of samples stored at 25 °C increased initially and then decreased, which may be attributed to the consumption of intermediate products (such as reductones and hydroxymethylfurfural) for melanoidin formation or their oxidative degradation, occurring at a rate exceeding their generation [54]. Samples stored at 35 °C exhibited a fluctuating trend in A_294_, characterized by an initial increase, followed by a decrease, and a subsequent rebound. In contrast, samples stored at 45 °C showed a marked increase in A_294_ in the later storage period. These results indicate that elevated storage temperatures promote the Maillard reaction, accelerating the formation of aromatic intermediates.

As shown in Figure 7d, the A_420_ values of all three groups of 3D-printed pork products demonstrated an overall increasing trend throughout the storage period. After 44 days of storage, the A_420_ values reached 126.51%, 149.51%, and 217.94% of the initial value, respectively, with statistically significant differences observed among the groups (*p* < 0.05). Higher storage temperatures resulted in greater A_420_ values, indicating that elevated temperatures promote the formation of advanced Maillard reaction products, particularly when the storage temperature exceeds 35 °C, where the rate of late-stage Maillard reaction increases substantially. Under higher storage temperatures, the polymerization of intermediate Maillard reaction products accelerates, thereby facilitating the generation of terminal melanoidins [53]. At any given storage time, A_420_ increased with rising storage temperature, a trend consistent with changes in product color. Notably, when the A_420_ value exceeded 0.6, the color of the 3D-printed pork products was no longer considered acceptable.

### 3.8. Pearson Correlation Analysis

**Figure 8:**
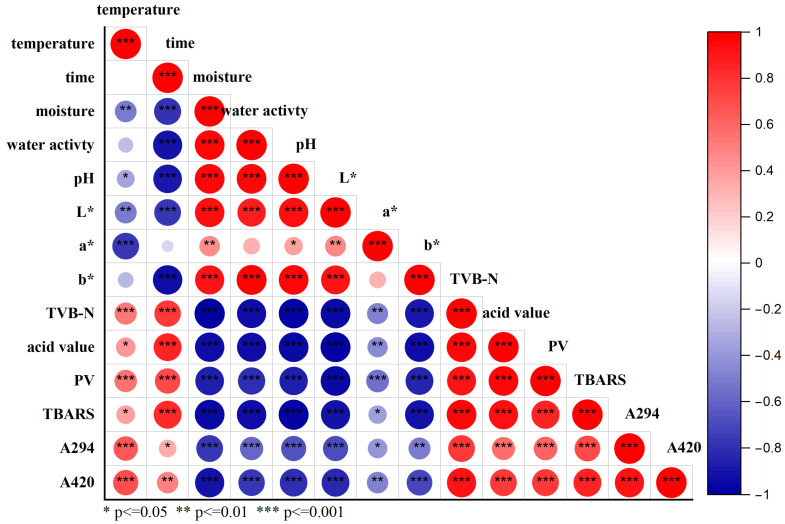
Pearson correlation analysis of temperature on the quality parameters of 3D-Printed Pork products during storage.

As illustrated in Figure 8, storage temperature showed a highly significant negative correlation with the a* value (redness) of the 3D-printed pork products (*p* < 0.001), while it exhibited highly significant positive correlations with TVB-N, peroxide value (PV), and the concentrations of Maillard reaction products (A_294_ and A_420_) (*p* < 0.001). These results indicate that higher storage temperatures adversely affect color quality and significantly promote lipid oxidation, Maillard reaction, and accumulation of its products, collectively impairing flavor and visual characteristics during storage.

Storage time was negatively correlated with moisture content, water activity, pH, and color parameters L* (lightness) and b* (yellowness). In contrast, it was positively correlated with TVB-N, lipid hydrolysis and oxidation indicators (acid value, TBARS, and PV), as well as Maillard reaction products (A_294_ and A_420_) (*p* < 0.05). Furthermore, a highly significant positive correlation was observed between acid value and lipid oxidation indicators (*p* < 0.001), while color parameters were significantly negatively correlated with the extent of the Maillard reaction (*p* < 0.05).

In summary, prolonged storage time and elevated temperature collectively contribute to the quality deterioration of 3D-printed pork products.

### 3.9. Changes in Volatile Flavor Compounds of 3D-Printed Pork Products During Storage

**Figure 9:**
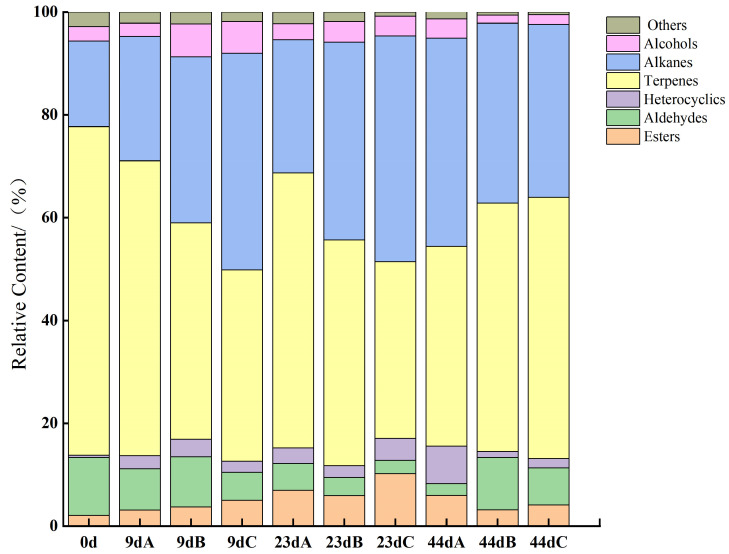
Effect of Storage time and temperature on the relative content of flavor compounds in 3D-Printed Pork Products. Note: Letters A, B, and C denote storage temperatures of 25 °C, 35 °C, and 45 °C; the storage time are 0d, 9d, 23d, 44d, respectively. 9d A, 9d B, 9d C denote 3D-Printed Pork Products storaged for 9d in 25 °C, 35 °C, and 45 °C, respectively.

A total of 233 volatile flavor compounds were identified in the 3D-printed pork product over the storage period (Figure 9), including 26 alcohols, 17 esters, 58 terpenes, 69 alkanes, 20 aldehydes, 29 heterocyclic compounds, and 14 compounds classified into other categories. Esters and alcohols contributed minimally to the overall aroma profile of the printed pork product. Hydrocarbons—specifically terpenes and alkanes—were the most abundant class of volatile compounds detected, a finding consistent with previous research by Deng et al. (2021) [55]. While most alkane-derived volatiles exhibit high odor thresholds and thus have limited influence on flavor, certain terpenoid compounds significantly enhance the sensory characteristics. Notably, compounds such as caryophyllene, D-limonene, zingiberene, and α-curcumene were present in considerable proportions, imparting distinct fruity and gingery notes. These flavor attributes are likely derived from seasoning ingredients including black and white pepper powders, as well as ginger powder, used in the marination process [55].

Aldehydes contributed significantly to the flavor profile of the printed pork product. During storage, the relative content of aldehydes detected in the samples exhibited a biphasic trend, characterized by an initial decline followed by a subsequent increase. Nonanal, hexadecanal, and phenylethanal were identified as common aldehydes across all 13 tested samples and played a pivotal role in flavor formation. Among these, nonanal—primarily derived from the oxidation of unsaturated fatty acids—imparts distinct oily and citrus-like notes, representing a major contributor to the aldehyde flavor compounds. Hexadecanal, generated through the oxidation of saturated fatty acids, contributes fruity aroma characteristics. Phenylethanal, a key odorant resulting from oxygen-involved Maillard reactions, exhibits a rose-like scent [55].

Heterocyclic compounds, largely formed either through the degradation of Maillard reaction intermediates (MRIs) or via Strecker degradation between dicarbonyls and amino compounds, are important volatile aromatic substances [55]. Most heterocyclic compounds confer roasted and meaty flavors. Both the variety and relative concentration of these compounds increased with higher storage temperatures. Furthermore, storage temperature was found to exert a more pronounced influence on the generation of heterocyclic compounds than storage duration.

Pyrazine compounds contributed distinct nutty and roasted aromas to the printed pork product. Samples stored at 45 °C exhibited increased formation of pyrazines in the advanced storage phase, likely due to the combined effects of elevated temperature and reduced water activity, which facilitated the generation of these flavor compounds [56].

Acid compounds, often produced through the re-oxidation of aldehydes and ketones under high-temperature Maillard reactions, were only detected in one sample (44dC) among the 13 tested groups after extended storage. The detected acids—including butanoic acid, 3-methylbutanoic acid, octanoic acid, and nonanoic acid—suggest that their formation requires prolonged exposure to high temperatures. These acids generally impart sharp, unpleasant odors that adversely affect the overall flavor profile.

Among ketones, 6-methyl-5-hepten-2-one was common to all samples and contributed fruity notes to the product. Piperitone, another ketone detected, originated primarily from seasoning ingredients used in the marination process.

### 3.10. Analysis of Key Flavor Compounds in 3D-Printed Pork Products During Storage

The contribution of volatile flavor compounds to the overall flavor profile of printed pork products depends not only on their concentration but also on their odor activity values (OAVs), as determined by their odor thresholds [57]. To clarify changes in key aroma components across different samples, Relative Odor Activity Value (ROAV) analysis was employed. A higher ROAV indicates a greater contribution of the compound to the overall aroma.

As summarized in Table 2, between 6 and 11 key volatile flavor compounds (ROAV ≥ 1) were identified in each of the 13 samples, comprising mainly aldehydes, terpenes, and heterocyclic compounds. Notable among these were nonanal, phenylethanal, D-limonene, zingiberene, and α-curcumene, which exhibited high ROAV values and were consistently detected across all samples. These six compounds are considered crucial contributors to the characteristic aroma of the product, imparting fatty, citrus-like, and fresh notes, and can be regarded as the dominant flavor substances.

Nonanal was identified as the most significant contributor to the overall flavor profile, imparting a pronounced fatty aroma that positively influenced the sensory characteristics of the product. (E)-2-Octenal and octanal were determined to be key flavor compounds in the early storage period, contributing fatty and fruity notes [58].

In sample 44 dC, 2,5-dimethylpyrazine and 2,3-diethyl-5-methylpyrazine were identified as critical volatiles, primarily providing roasted and caramel-like odors.

Phenylethanal, caryophyllene, D-limonene, zingiberene, α-curcumene, and β-sesquiphellandrene exhibited an initial increase followed by a decrease in ROAV values during storage, peaking at day 23. Higher storage temperatures correlated with increased ROAV values for these six compounds. Their positive contribution to the aroma profile of the 3D-printed pork product suggests that elevated storage temperatures, within a certain timeframe, can enhance the flavor quality.

## 4. Conclusions

Pea protein significantly influences the printability of pork-based materials, with a mass ratio of pork to pea protein of 5:1 (20% of pea protein inserted in pork paste) yielding the optimal printing performance. From a macroscopic perspective, the incorporation of pea protein notably enhanced the storage modulus (G′), loss modulus (G″), and gel strength of the pork paste, with these properties increasing proportionally with higher protein concentrations. The consistent observation that G′ exceeded G″ across all formulations indicates solid-like behavior, suggesting that the addition of pea protein improves the structural stability of 3D-printed pork products and reduces the risk of deformation or collapse. Pea protein induced significant alterations in the relaxation times and relative peak areas of the pork gel, reflecting modified water distribution states within the system. These changes directly impacted the printing behavior. Such structural reorganizations at the molecular level correlate with the observed macroscopic improvements in printability and product integrity.

During the storage of 3D-printed pork products, elevated storage temperatures adversely affected flavor quality, which can be attributed to accelerated lipid oxidation and Maillard reaction pathways, leading to the accumulation of associated volatile and non-volatile compounds. Furthermore, storage time was negatively correlated with moisture content, water activity, pH, and color parameters L* and b*. In contrast, a significant positive correlation (*p* < 0.05) was observed between storage duration and the accumulation of total volatile basic nitrogen (TVB-N), lipid hydrolysis and oxidation products (as indicated by acid value, TBARS, and peroxide value), and Maillard reaction intermediates (absorbance at 294 nm and 420 nm). A highly significant positive correlation (*p* < 0.001) was identified between acid value and lipid oxidation indicators, while color parameters were significantly negatively correlated (*p* < 0.05) with the extent of the Maillard reaction.

A total of 233 volatile flavor compounds were detected, with terpenes and alkanes representing the most abundant classes. Among these, 19 key aroma compounds were identified, with nonanal, phenethyl alcohol, D-limonene, zingiberene, and α-curcumene contributing most significantly to the overall flavor profile.

In summary, elevated storage temperatures and time leading to a notable deterioration in the storage quality of the 3D-printed pork products. The shelf life of 3D-printed pork products, when stored without preservatives, is limited to 44 days at a recommended maximum temperature of 35 °C.

## Figures and Tables

**Table 1:** The T_2_ and S_2_ values of 3D-printed pork products.

		T_21_/ms	T_22_/ms	T_23_/ms	S_21_/%	S_22_/%	S_23_/%
ratios of pork to pea protein	2:1	0.95 ± 0.14 ^b^	21.47 ± 1.05 ^f^	204.91 ± 0.00 ^f^	10.05 ± 0.66 ^a^	86.95 ± 0.79 ^e^	3.00 ± 0.13 ^a^
	3:1	1.77 ± 0.26 ^a^	25.53 ± 0.00 ^e^	252.35 ± 0.00 ^e^	9.99 ± 0.17 ^a^	88.09 ± 0.24 ^d^	1.92 ± 0.07 ^b^
	4:1	1.18 ± 0.29 ^b^	29.33 ± 0.00 ^d^	289.94 ± 0.00 ^d^	8.11 ± 0.11 ^b^	90.46 ± 0.06 ^c^	1.43 ± 0.05 ^c^
	5:1	1.34 ± 0.20 ^ab^	31.44 ± 0.00 ^c^	333.13 ± 0.00 ^c^	7.93 ± 0.5 ^bc^	90.98 ± 0.17 ^bc^	1.09 ± 0.02 ^d^
	6:1	1.25 ± 0.06 ^b^	33.70 ± 0.00 ^b^	382.75 ± 0.00 ^b^	7.20 ± 0.31 ^cd^	91.90 ± 0.30 ^ab^	0.90 ± 0.01 ^e^
	7:1	1.17 ± 0.17 ^b^	36.12 ± 0.00 ^a^	425.01 ± 20.86 ^a^	7.00 ± 0.20 ^d^	92.29 ± 0.21 ^a^	0.71 ± 0.01 ^f^

Note: Different superscript letters indicate significant differences (*p* < 0.05) among values for the same parameter.

**Table 2:** Key flavor compounds and the ROAV values in 3D-printed pork products.

Key Flavor Compounds	Threshold/(μg/Kg)	ROAV									
		0 d	9 d A	9 d B	9 d C	23 d A	23 d B	23 d C	44 d A	44 d B	44 d C
Nonanal	3.5	100	100	100	100	100	100	100	100	100	100
n-Hexanal	5.0	12.0	4.7	9.2	11.8	10.9	9.1	—	1.6	—	7.0
n-Heptanal	3.0	0.6	—	0.4	—	—	0.6	—	0.2	—	0.4
Phenyleacetaldehyde	2.0	5.4	15.6	14.3	15.7	20.6	37.1	49.6	3.3	6.4	9.2
(E)-2-Octenal	3.0	1.9	4.7	7.2	—	6.8	—	—	—	—	—
n-Octanal	47.0	—	1.6	1.9	2.2	—	—	—	—	—	—
2,5-Dimethylpyrazine	36.0	0.3	—	2.1	—	4.0	5.9	6.7	0.6	1.6	6.0
2,5-Dimethyl-3-ethylpyrazine	200.0	—	0.1	0.2	0.1	0.2	0.4	0.3	—	—	—
2-Ethyl-6-methylpyrazine	250.0	—	—	—	—	—	—	0.2	—	—	0.1
2,3-Diethyl-5-methylpyrazine	5.0	—	—	—	—	—	—	—	—	—	4.6
2-Methylpyrazine	250.0	—	—	—	—	—	—	—	—	—	0.2
2-Methylpyridine	14.0	—	—	—	—	—	—	—	—	—	—
β-Caryophyllene	1 500	0.7	1.1	0.8	1.4	1.8	2.2	2.4	0.5	0.9	0.6
D-Limonene	45.0	8.9	9.4	7.5	11.9	18.5	28.9	17.5	5.5	9.6	8.4
Zingiberene	20.0	8.7	12.4	9.1	16.1	24.1	23.8	29.9	5.9	10.8	10.5
α-Curcumene	15.0	10.6	18.2	13.8	24.1	33.6	33.0	40.7	8.0	16.4	16.8
β-Phellandrene	0.2	0.4	0.6	0.5	0.8	1.2	1.2	1.6	0.3	0.6	0.6
Linalool	3.8	—	—	—	—	—	—	—	1.6	—	8.2
Myristicin	30.0	0.3	0.4	0.3	0.6	0.8	0.9	1.7	0.2	0.4	0.7

Note: Letters A, B, and C denote storage temperatures of 25 °C, 35 °C, and 45 °C, respectively.

## Data Availability

The original contributions presented in the study are included in the article, further inquiries can be directed to the corresponding author.
